# Health in All Networks Simulator: mixed-methods protocol to test social network interventions for resilience, health and well-being of adults in Amsterdam

**DOI:** 10.1136/bmjopen-2025-100703

**Published:** 2025-04-25

**Authors:** Jiri Kaan, Ciska Ulug, Kristina Thompson, Yara Khaluf, Annemarie Wagemakers, Spencer Moore

**Affiliations:** 1 Health & Society, Wageningen University, Wageningen, The Netherlands; 2 Information Technology, Wageningen University, Wageningen, The Netherlands

**Keywords:** Health, PUBLIC HEALTH, Obesity, Social Interaction, Social Support

## Abstract

**Background:**

Social networks are an important, although overlooked, component of community-based health promotion. Advances in social network research have highlighted different social network intervention (SNI) strategies to improve community-based health promotion. The aim of this project is to collaborate with community and policy stakeholders to explore how to best apply these SNI strategies to improve the resilience, health and well-being of adults in Amsterdam, and more broadly in the Netherlands.

**Methods and analysis:**

To this end, we will collaboratively develop an intervention planning tool called the ‘Health in All Networks Simulator (HANS)’. This tool will be capable of virtually testing different SNI strategies and forecasting their possible impact on resilience, health and well-being. Taking a mixed-methods approach consisting of a combination of interviews, group model building workshops and agent-based modelling with members of two communities in Amsterdam and policy stakeholders, we will foster a shared learning process while ensuring ownership and relevance of HANS to ongoing community-based health promotion practice.

**Ethics and dissemination:**

The research project has been approved by the research ethics committee of Wageningen University (approval numbers: 2024-039; 2024-226). HANS will be shared directly with stakeholders. The results will be made available to the public via open-access publications and conferences.

**STRENGTHS AND LIMITATIONS OF THIS STUDY**

- Including the perspectives of community and policy stakeholders will contribute to the relevance and ownership of the intervention planning tool.
- Using visual and interactive methods during the workshops may help improve understanding of complex systems by stakeholders.
- A participatory modelling approach could help better reflect the lived realities of the participating neighbourhoods.
- One limitation could be that participants’ mental models are incomplete or subjective, which leads to biased results.
- Another potential limitation is that the conceptual models developed during the participatory workshops held in two neighbourhoods may not converge.

## Introduction

 Social networks, the patterns of social ties existing among a set of actors, profoundly influence health and well-being.^1^,^6^ Despite their profound influence, social networks are often under-theorised or overlooked when designing, implementing or evaluating community-based health promotion or health promotion in general. For example, only 3.7% of childhood obesity prevention efforts have explicitly targeted social networks, with most focusing solely on educating children.^7^ Targeting people without accounting for their social networks has repeatedly shown limited and inconsistent results across diverse populations.^5 8 9^ Furthermore, leveraging social networks has been shown to improve health behaviours and outcomes, as demonstrated by several reviews.^510^,^12^

Social network interventions (SNIs) explicitly aim to improve health and well-being by leveraging social networks and their characteristics.^11^ SNIs can help accelerate behaviour change, disseminate information, generate social influence and achieve other desired outcomes within individuals, communities and populations.^12^,^14^ Advances in social network research have highlighted different SNI strategies: (1) targeting individuals with specific network characteristics (individual); (2) targeting specific people or groups (segmentation); (3) activating the network to form novel connections (induction); and (4) modifying the network (alteration).^11^ A meta-analysis indicates that SNIs are associated with better health outcomes and behaviours compared with controls, with the strongest evidence for targeting individuals based on certain network characteristics.^10^ However, because community-based health promotion practice often involves multiple active components (eg, increasing physical activity), it remains challenging to isolate the specific contribution of social networks to health and well-being.

To address these challenges, complexity science approaches are increasingly used in public health^15^,^18^ to understand how SNIs can improve health and well-being.^19^,^21^ These approaches study how systems with many interconnected parts behave as a whole, focusing on the interactions, relationships and emergent properties that arise from these dynamics.^22 23^ For example, researchers simulated how social network characteristics could be used to select influence agents among schoolchildren, who were then trained to encourage peers to be more physically active.^19^ Similarly, simulations provided insights into whom should receive a weight loss intervention within a social network.^20^ Both computational models demonstrated that targeting individuals based on their network properties outperformed conventional strategies, such as focusing on high-risk individuals. In short, these models facilitate insightful experimentation with various scenarios that would be difficult to implement in the real world, providing a unique tool for community and policy stakeholders to assess potential outcomes before making decisions.^24^

Community and policy stakeholders can be included in the development of computational models through participatory modelling. This approach fosters shared learning, improves the likelihood of implementation and elicits mental models from stakeholders that are crucial for the development of computational models.^25^,^27^ Mental models are relatively long-lasting internal conceptual representations of external systems that people use to understand and interact with them.^28^ In other words, it is assumed that community and policy stakeholders possess structural knowledge of the system and that making these mental models explicit leads to more precise models.^29^ Aligning mental models in a group may additionally lead to greater model precision of real-world dynamics while providing stakeholders with an overview of the system and opportunities to induce change.^25^,^27^

Stakeholder involvement helps to model real-world dynamics as perceived by stakeholders, contributing to its face validity. However, validation in computational modelling is a complex and much-debated topic,^24^ particularly due to the inherent simplifications of real-world systems, which create challenges in balancing realism with computational tractability.^30 31^ Validation is not a one-size-fits-all process; it involves multiple steps, such as calibration, sensitivity analysis and comparison to empirical data, all of which require careful consideration depending on the complexity of the model. While participatory validation is valuable, and engaging in the process of participatory modelling may be sufficient in itself to initiate collective action towards solving a problem,^32^ over-reliance on this method may come at the expense of neglecting other forms of model validation, such as comparing model outcomes with empirical data or testing the model’s predictive power.^33^ Given that few studies report validation techniques beyond participatory methods, it is an open question how participatory modelling compares to other validation techniques in improving the accuracy and validity of computational models used to predict health and well-being.^33^

### Research objective

This research proposal is part of the research project ‘Health in All Networks Simulator (HANS)’. The project runs from June 2023 to June 2027. The main goal is to cocreate a computational model with community and policy stakeholders that can be used to forecast the impact of SNIs on resilience, health and well-being of adults in Amsterdam. Together with policy stakeholders and members of two communities in Amsterdam, we aim to gain insight into the real-world dynamics, while providing stakeholders with an overview of the system and opportunities to induce change. At last, we aim to investigate how participatory modelling contributes to the accuracy and validity of computational models in forecasting health and well-being.

## Theoretical framework

**Figure 1:**
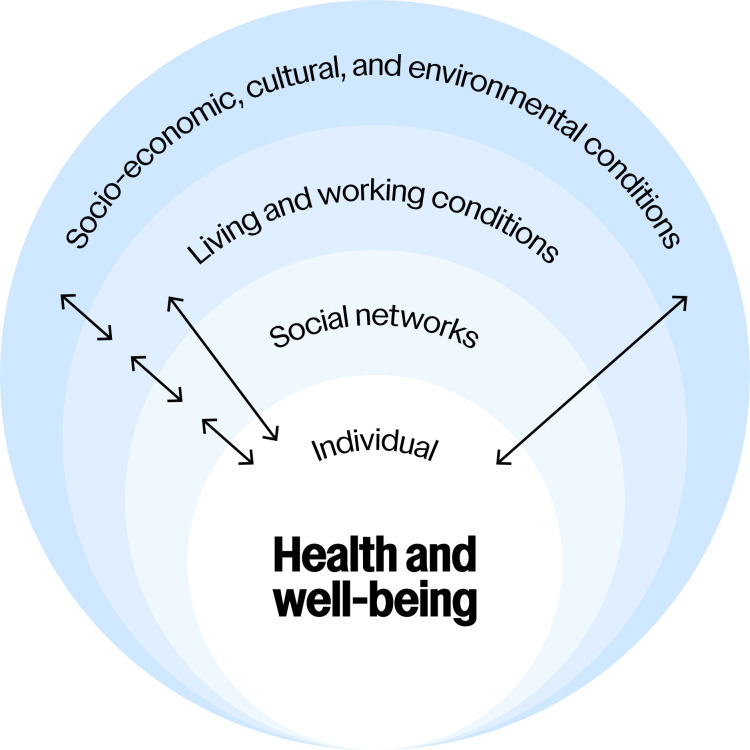
Health and well-being emerge from interactions in a multilevel system (inspired by Dahlgren and Whitehead^34^).

We conceptualise health and well-being as emergent properties that arise from dynamic, multilevel processes within a complex system.^9^ Emergence refers to the properties of a complex system that cannot be predicted solely by its individual components or their sum.^9^ In this view, health and well-being cannot be explained by isolated components (eg, individual action) but are shaped by a range of interacting components (eg, interactions in social networks) across levels.^9 22^ In line with this, the individual is assumed to be nested within a multilevel system consisting of social networks, living and working conditions, and socioeconomic, cultural and environmental conditions (ie, structural conditions) that shape health and well-being^34^ (figure 1).

Rather than solely targeting the individual within this multilevel system, we aim to target social networks to improve health and well-being. Social capital has been viewed as a key mechanism linking social networks to health outcomes.^35^ Social capital refers to the resources that are accessible through social networks.^36^ More social capital is not always better; certain types of social capital can be harmful for health and well-being.^37^,^40^ For instance, research on obesity and social networks shows that obesity tends to cluster in social networks, possibly by shaping down levelling norms and behaviours.^41^ People with more diverse ties and greater access to resources, however, tended to have a lower risk of adiposity and obesity.^42^ Having diverse and resourceful networks has been viewed as a defining element of social resilience.^43^ Social capital may foster resilience by enabling individuals and communities to support each other or access intracommunity or extracommunity resources.^44^ Hence, social capital serves both as a mechanism and an indicator of resilience in the relationship between social networks and health and well-being.

**Figure 2:**
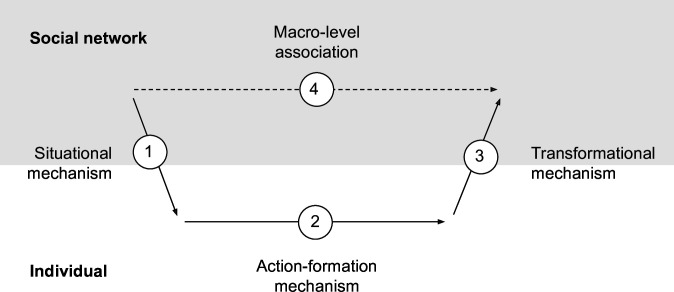
A mechanistic approach to embed the individual in social networks (inspired by Hedström and Ylikoski^45^).

To more precisely explain how social capital may foster resilience in social networks and drive health and well-being, a reliable approach may be to identify and define the (1) situational, (2) action-formation and (3) transformational mechanisms^45^ (figure 2). Situational mechanisms are defined as the mechanisms through which a person’s interaction with their immediate environment leads to the perception and choice of action alternatives.^46^ Social networks play a significant role by providing access to various psychosocial resources that enable or constrain action alternatives.^47^ Action-formation mechanisms are the mechanisms by which people may initiate a specific action in response to perceived alternatives and motivations.^45^ For instance, if people are motivated to seek help and perceive that their social network provides access to social support, they may act towards that goal. Less is known, however, about the transformational mechanisms that refer to how people, through their actions and interactions, may generate various intended and unintended health and well-being outcomes.

In summary, we embrace complexity. By conceptually embedding the individual in social networks with the help of situational, action-formation and transformational mechanisms to explain macrolevel associations, we aim to contribute to the need for consilience between the social determinants of health and individual health behaviour change, and potentially find precise explanations for the persistence of health inequalities.^48 49^ Overall, the theoretical framework serves as a starting point for the development of an intervention planning tool that can test SNIs to improve the resilience, health and well-being of adults in Amsterdam.

## Methods and analysis

### Study design

**Figure 3:**
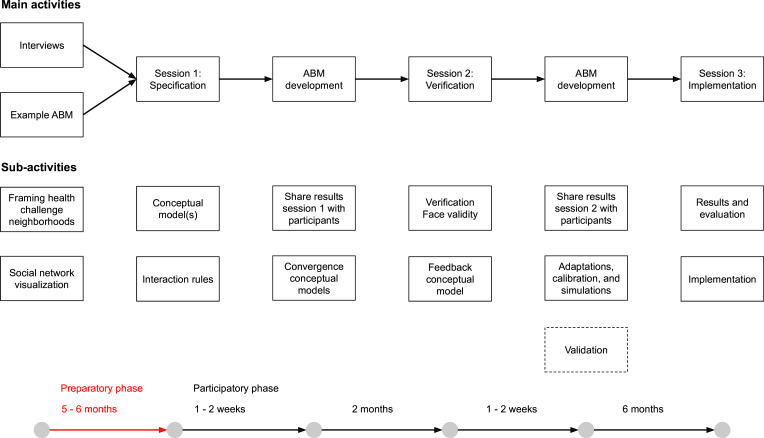
Timeline for group model building (GMB) sessions and agent-based model (ABM) development.

To develop an intervention planning tool—HANS—capable of testing relevant SNIs to improve the resilience, health and well-being of adults in Amsterdam, a mixed-methods study design will be used. The project consists of a preparatory phase and a participatory phase that are planned to take place from September 2024 to December 2025 (figure 3). The project is currently in the preparatory phase, which includes conducting interviews with neighbourhood residents, planning participatory workshops and developing an example computational model to demonstrate a complex systems approach during the workshops. During the participatory phase, three workshops will be held in each neighbourhood, with the intervention planning tool being cocreated and developed by the researchers in between workshops.

### Patient and public involvement

Community and policy stakeholders are involved in the preparatory and participatory phases of the research project. For instance, they will be asked to identify who should be interviewed and invited to the workshops and sometimes will be asked to participate themselves. In addition, their input will be sought throughout the duration of each workshop. During the interviews and workshops, we will decide together the health challenge to focus on in each neighbourhood based on the needs and experiences of the participants. Furthermore, we will decide together which intervention scenarios to investigate in relation to ongoing community-based health promotion practice. The research question(s) and outcome measures may vary accordingly. Participants and stakeholders will receive information and results in a format that meets their specific requirements.

### Preparatory phase

#### Interviews

In preparation for the participatory modelling sessions (see below), one of the coauthors will conduct in-depth scoping interviews with neighbourhood residents, asking about health challenges existing in their neighbourhoods networks. The interviews are additionally designed to provide insight into the psychosocial mechanisms linking the social networks to the individual,^47^ thereby providing essential input for the earlier mentioned situational mechanisms (figure 2).

The focus of the interviews and modelling sessions will be in two neighbourhoods in Amsterdam, where community partners are located. Both partners are involved in hosting a ‘neighbourhood restaurant’ (‘*buurtrestaurant*’), which will serve as an access point to the neighbourhood residents. The coauthor will regularly attend meals at the restaurant, in order to engage in more informal conversations, better understand the (social) environment, build trust with participants and ultimately recruit residents for interviews and the participatory modelling sessions.

Interview participants will be recruited based on the criteria that they live in one of the focus neighbourhoods and are willing to participate. We will aim for 10 interviews per neighbourhood, or until saturation is reached, and no new information emerges.^50^ This will be determined through discussions with the research team and community partner, once consensus has been reached on the health challenge to be addressed in the participatory modelling sessions.

In order to better understand residents’ neighbourhood networks, a visual mapping activity will be implemented in the interviews, based on Hogan *et al*.^51^ Similar to ‘name generator’ exercises, participants will be first asked to name neighbourhood contacts and then instructed to categorise these names and arrange them in concentric circles, based on closeness. The names provided during this activity will be converted to numbers, and the (individual) social networks maps will be combined in a digital format. This mapping activity is otherwise done completely analogue, using cardboard cut-outs and post-its. The low-tech nature has several advantages, such as being accessible, creative and fun, as well as less intimidating and susceptible to technology failures associated with digital networking activities.^51^ Residents will be asked about health (challenges), the role of health in the network (eg, how it supports a healthy environment and prevalent health challenges in the network) and potential interventions residents find helpful (eg, the role of the neighbourhood restaurant) (see the online supplemental interview guide in appendix 1). Combining the visualisation activity with an interview allows the researcher to pose open-ended questions around the network and use it as a prop to dig deeper into potential health challenges.^52^ As a result, this method has been especially praised for working with more sensitive topics.^53^

Interview transcripts will be transcribed and coded using Atlas.ti coding software. The analysis will follow thematic analysis^54^ and will be predominantly inductive and otherwise based on psychosocial mechanisms linking social networks to the individual.^47^

During the interviews, residents will also be invited to participate in the participatory modelling sessions, as the health challenges described will serve as a basis for the sessions. Specific benefits for participation will include strengthening the community, acquiring insight into the neighbourhood’s health dynamics and gaining ownership of the health challenge.

#### Example computational model

In addition to the interviews, an example computational model will be developed in preparation for the participatory modelling sessions. Complexity science offers several computational methods to better understand complex phenomena such as health and well-being.^18^ In this project, we will use agent-based models (ABMs) to study health and well-being. ABMs enable researchers to model individuals (agents) and their actions and interactions with each other and their environment, thereby simulating how complex system behaviours emerge.^31 55^ One of the key advantages of ABMs is their flexibility in modelling heterogeneous populations, allowing us to simulate how differences within and between neighbourhoods affect resilience, health and well-being.

In our example ABM, we apply the theoretical framework outlined above, meaning that agents are embedded within structural conditions, such as social networks (figure 1). These conditions may influence agents through situational mechanisms (figure 2), which provide opportunities for action. To model how agents act and adapt to these opportunities—or lack thereof—we may use reinforcement learning and/or Bayesian inference-making to formalise the action-formation mechanisms.^56^,^58^ With this approach, we can model the transformational mechanism—that is, the interactions between agents and structural conditions that give rise to resilience, health and well-being—and how SNIs impact these.

The example ABM and, ultimately, HANS will be programmed in Python with the Mesa and NetworkX packages. To estimate the potential effect of SNIs, multiple simulations (eg, 1000 times) will be run to produce time series data with an 80% CI around health and well-being variables in scenarios with or without SNIs. Furthermore, the degree to which resilience mediates or moderates the relationship between the social network and outcomes related to health and well-being will be analysed. Data will be analysed at average population levels, but also grouped by, for example, socioeconomic status/neighbourhood/communities to gain insight into health inequalities/inequities.

It is important to note that at this stage, the example ABM is mostly theoretical. In HANS, resilience will be operationalised at the collective level in terms of neighbourhood social capital consisting of measures of generalised trust, social cohesion and network diversity. Because our community partners have neighbourhood restaurants, we will focus health outcomes on diet-related, chronic conditions such as diabetes and obesity. Well-being will be operationalised holistically with aspects of physical, mental and social well-being as measured by the 12-Item Short-Form Health Survey. However, the operationalisation may change as a result of the interviews and the ensuing participatory modelling workshops, depending on the needs of policy and community stakeholders.

### Participatory phase

#### Group model building (GMB)

We will host participatory modelling workshops, specifically GMB, in collaboration with community and policy stakeholders. GMB is a process in which stakeholders are deeply involved with the researchers to collaboratively build and analyse a computational model of complex systems.^25^ The workshops will take place over three sessions in Amsterdam, with each session ending with a dinner at the neighbourhood restaurant (table 1).

**Table 1:** Agenda and scripts for group model building sessions

Session 1 (120 min)	Session 2 (90 min)	Session 3 (90 min)
Walk in and welcome	Walk in and welcome	Walk in and welcome
Concept model	Model review	Model review
Icebreaker	Simulations	Simulations
Variable elicitation	Break	Break
Break	Small group review	Discussion model
Creating a causal loop diagram from variable list	Big group review	Tutorial and implementation
Next steps and closing	Action ideas	Next steps and closing
	Next steps and closing	

The GMB sessions will take place in the two neighbourhoods, where the community partners are located. Participants will ideally include a combination of neighbourhood residents (eg, those recruited during interviews and neighbourhood restaurants), policymakers and practitioners working on topics of health and well-being in the city. Practitioners could include municipal workers, neighbourhood initiatives and academics. We will aim for 10–15 participants in each session, based on recommendations from the literature.^32^ Inclusion criteria therefore include that participants are working on topics related to health and well-being and/or are involved in the target neighbourhood and can therefore act as an ‘opinion leader or interpreter within their community’ [Hovmand PS,^32^,p26]. We will also aim to balance out these voices and include a diversity of participants, which means at least one resident, one civil servant, one neighbourhood initiative, one scholar and no more than three participants from each category. Community partners will serve as gatekeepers to invite potential participants.

#### Session 1

The goal of the first session is to collaboratively design a conceptual model. The example ABM and the details derived from in-depth scoping interviews will be used to design a preliminary conceptual model. The project will be briefly introduced at the beginning of the session, along with the background of systems thinking and the initial conceptual model using the script ‘concept model’. The script will be explained from the frame of reference of ABMs rather than a system dynamics model for which the script was originally created, similar to Frerichs *et al*.^59^ ABMs simulate the system using decentralised agent actions and interactions with other agents and/or their environment (‘bottom-up’) rather than stocks and flows of aggregated variables (‘top-down’).^60^ A benefit is that the frame of reference of ABMs may be more intuitive to understand for neighbourhood residents.^59 61^

Next, we will conduct a short ice-breaker to prepare participants for a fun, interactive session. Then we will start with the variable elicitation script where participants will be prompted with the question ‘What are some things that help or make it harder to (*identified health/well-being challenge*)?’.^59 62^ Special emphasis will be placed on agent characteristics that may influence their interactions with other agents and/or the environment, or vice versa. Finally, relationships between the identified variables relevant to the health/well-being challenge will be mapped by using the ‘creating causal loop diagram from a variable list’ script using drawing conventions for participatory modelling for ABMs,^61^ yielding a comprehensive conceptual model. Concluding remarks will be based on the ‘next steps and closing’ script. All scripts are publicly available here.

### Intersession model development 1

The results of the first GMB session will be shared with the participants as soon as possible. With the information and insights from the first GMB session, a first version of HANS will be developed. At this stage, the ABM is not yet data-driven to keep the time between GMB sessions as short as possible (figure 1). An important design aspect is that the ABM should visually resemble the created conceptual model as much as possible to help participants recognise and understand the ABM. Moreover, an intuitive and interactive interface can help facilitate that participants test the ABM with varying parameter configurations themselves so that they can provide better feedback as to whether the ABM is plausible and/or realistic according to their lived experiences.^61^

#### Session 2

In the second session, the conceptual model from the first session will be shared again and initial simulations in HANS will be shown. Depending on the size of the group, stakeholders will be randomly divided into smaller groups and encouraged to validate and/or critique the conceptual model and the simulations, identifying areas for improvement and ensuring that specifications are met. Participants are asked (1) how similar the behaviours of agents in the model are compared with the real world and (2) how relevant the simulated SNIs are to the identified health/well-being problem and ongoing practice. Afterwards, participants come back together in a big group and share their answers to the questions. This is done to ensure that the model accurately represents what it is supposed to represent, contributing to its face validity.^30^ The session will conclude by brainstorming potential formats for outputs, to make the model useful, interesting and fun for community residents and stakeholders, for example, a serious game.^63^

### Intersession model development 2

The results of the second GMB session will be shared with the participants as soon as possible. Based on the input received, HANS will be modified in between sessions and become data-driven. This may include creating a synthetic population of Amsterdam using Census data from the Central Bureau of Statistics and the SynthEco framework,^64^ using Geoscience and Health Cohort Consortium to characterise the physical and built features of Amsterdam neighbourhoods,^65^ combining social network data from interviews with social network data of Amsterdam residents using CBS data^66^ and using Healthy Life in an Urban Setting cohort data for health, well-being and social data of ethnic populations in Amsterdam.^67^ If feasible, further validation will include comparing model outcomes to empirical data, exploring how GMB workshops contribute to validation techniques other than face validity.

After calibration and/or validation, we will simulate three SNIs according to the needs of policy and community stakeholders to forecast effects on health and well-being. The data analysis approach will be comparable to that used in the ABM example. Depending on the needs of community and policy stakeholders, additional data analyses will be considered. HANS will allow us to consider the possible spillover effects of an SNI in one neighbourhood on the resilience, health and well-being of others. The modelled transformational mechanisms may lead to intended as well as unintended spillover effects between mechanisms. Taking these spillover effects into account will provide a more complete picture of the overall population-level effects of SNIs and whether these changes are associated with changes in health inequalities

#### Session 3

In the final session, we will review the final conceptual model and share the results of the simulations of the final model. The implications of the simulations will be thoroughly discussed and what it may mean for practice. In a tutorial, community and policy stakeholders will be taught how to use the model, to ensure that ownership can be properly transferred to the participants. The tutorial could highlight how stakeholders might use it for policy and intervention planning and how to further implement the model in their decision-making. The form of output discussed in the second session will be prepared between the sessions and also presented to participants. This output will be more suitable for wider audiences, such as neighbourhood initiatives and residents.

## Ethics and dissemination

The research project was approved by the research ethics committee of Wageningen University on 3 May 2024 (2024-039) and the additional interviews were approved on 12 December 2024 (2024-226). Informed consent will be asked at every stage of the project that involves participants and participants will be informed about the goal and the activities of the study. Written consent will be obtained, and otherwise oral consent when necessary. Additionally, participants may withdraw from research activities at any time without having to provide a reason why. Participation is voluntary, but if participants attend all three workshops they will receive a gift voucher of €50 to compensate for their time. Additionally, travel and/or parking costs will be reimbursed and dinner after the workshops will be provided.All publications resulting from the research of our project will be made available via open access and using the Creative Commons Attribution. Furthermore, the results from the group model building workshops will be shared directly with the community and policy stakeholders. Documentation and the model code of HANS will be fully open source and available on appropriate sites (eg, GitHub).

## Supplementary material

10.1136/bmjopen-2025-100703 online supplemental file 1
