# Supplementary material for: Health in All Networks Simulator: mixed-methods protocol to test social network interventions for resilience, health and well-being of adults in Amsterdam
Source: BMJ Open. 2025 Apr 25;15(4):e100703. doi: 10.1136/bmjopen-2025-100703 (PMC12035472; doi:10.1136/bmjopen-2025-100703)
Supplement: online supplemental file 1 [file bmjopen-15-4-s001.docx]

***Interview Guide***

**Introduction**

- Can you begin by introducing yourself and telling something about how you came to live in this neighborhood?
  - Probe: what year?
  - What characteristics does this neighborhood have? What do you like about this neighborhood?
  - How are you involved in this neighborhood (e.g. volunteer work)?

**Health: What are the health opportunities and challenges, as defined by residents?**

As I mentioned, we are specifically interested in health, and how it is perceived among people in the neighborhood.

- What makes you feel (un)healthy?
  - Can you explain?
  - Can you give an example of when you feel (un)healthy?
  - How would you define health?

**Social network**

I would also like to ask about your local contacts because we are interested in the role of social networks in the neighborhood.

To better understand your social networks, and how they are related to health, I would like to visualize them with with you. I will ask you to list the names of your social contacts, and then I want to distinguish how close or not close they are to you: “very close” and “somewhat close”. Very close (green post-its) are people with whom you discuss important issues, with whom you keep in touch regularly, and who are there for you when you need help. Somewhat close (pink post-its) are people who are more than casual acquaintances, but still not very close.

I would like you to start with the names that are very close to you first and then write down the names that are somewhat close to you. It's also fine to switch between the two if you like.

*(perform exercise and write down contacts)*

Thank you for listing all the names. Now I want to label these names according to different role categories. These are:

1. Close relatives/relatives? (husband, wife, father, mother, brother, child)
2. Other relatives (uncle, aunt, etc.).
3. Friends?
4. Neighbors (with anyone?)
5. People with whom you work or go to school
6. People you only know online
7. People from different organizations (clubs, church, sports teams)
8. Friends not included above
9. Others

It's also fine if someone appears in more than one category. If you are reminded of someone else, feel free to add more names to the lists as well.

*(categorize the names)*

Okay, great. Now let’s organize these names into four different circles. The circle closest to the middle are the people you feel closest to, and the circle most outward are the people you feel less close to. We will start with the Very Close names you came up with and then the Somewhat Close names. I would ask you to place people who know each other close together. You may always reassess people in how “close” people are to you, so you are not bound by your initial classification.

Next, I would like you to indicate who knows each other by drawing circles or lines connecting the names that know each other.

Now I have a few questions I want to ask about the social network we've created.

You have identified groups of people. Can you describe these further?

- How do these people know each other?
- What does the interaction look like?
- How are they bonded?
  - Are there certain characteristics they have in common?
  - For example: cultural background, demographics (age, education)
  - Are there certain activities?

Of all the contacts you have mentioned…:

- Is there anyone who motivates you to be healthy or encourages healthy behaviors?
  - Probe: Connected to neighborhood
  - Probe: Related to social network groups
- How is the (un)health of people around you, in your neighborhood/social network?
  - Are there any health problems that are especially prevalent among these people?
  - Probe: Are there any particular challenges that are going on here, in your neighborhood?
  - Probe: Where might these come from?
  - Probe: Are actions already being taken to counter these? Are these actions effective?
  - Probe: If not, what can be done?
- I have heard that social isolation is a problem experienced by some residents in this neighborhood.
  - What do you know about this?
    - Is there anything that helps you overcome this challenge? If so, what?
  - Have you heard about this from other contacts in your area?
    - For example, friends, family, neighbors, colleagues, etc.
  - Would any of these contacts be interested in discussing this issue further?
- What do social networks / your local contacts mean to you in this neighborhood?
  - Do you get support from these people? How?
    - Probe: Examples?
    - Probe: How does social network contribute to health?
    - Probe: What does it take to get there?
- You also frequent the neighborhood restaurant. Why did you start going there?
  - Why do you keep coming?
  - What do you like about it?
    - Probe: psychosocial mechanisms (social support, engagement, access to resources)
    - Are you connecting with new people?
    - Do you feel more involved in the neighborhood?
    - Do you connect with opportunities that you would not otherwise get?
- When do you not come to the restaurant? And why?
- Do you eat differently at the neighborhood restaurant than you do at home?
  - If yes, how does the food differ?
  - Probe: composition, quantity, quality, culture, health, taste
  - Have you started eating differently at home since eating at the neighborhood restaurant?
- I also noticed that this is quite a culturally diverse neighborhood, which is also reflected in the neighborhood restaurant with the different cuisines served there.
  - How did you experience that?
  - Probe: Have you learned anything? Gained different perspectives?
  - Do you come in contact with people from different social and cultural groups?
- Do you know people who could benefit from also coming to the neighborhood restaurant?
  - Would any of these contacts be interested in discussing this topic further?

**Reciprocity**

In this survey, we also want participants to get something out of it.

- Is there anything you would like to know about health in this neighborhood? What exactly? Give examples
- Is there anything you would like to see come out of this? Give examples.
  - What type of output would be helpful to you?
  - E.g. more contacts with neighbors, more sources of information about healthy lifestyles, funding/grants for neighborhood programs to promote health.
  - I can't promise anything, but this will help us focus our efforts.

**Closing**

Finally, there are some demographic questions I would like to ask:

- Question: demographics: year of birth? Cultural background? Education level?

Those were all the questions I had for you. Do you have anything else to add, or questions for me?

Thank you again for your time. I really appreciate it. If you know of anything else to add, or if you have any questions later, please don't hesitate to get in touch.
