# Supplementary material for: Health in All Networks Simulator: mixed-methods protocol to test social network interventions for resilience, health and well-being of adults in Amsterdam
Source: BMJ Open. 2025 Apr 25;15(4):e100703. doi: 10.1136/bmjopen-2025-100703 (PMC12035472; doi:10.1136/bmjopen-2025-100703)
Supplement: Supplementary file 2 [file bmjopen-2025-100703.reviewer_comments.pdf]

PEER REVIEW HISTORY

BMJ Open publishes all reviews undertaken for accepted manuscripts. Reviewers are asked to complete a checklist review form and are provided with free text boxes to elaborate on their assessment. These free text comments are reproduced below.

ARTICLE DETAILS

Title (Provisional)

Health in All Networks Simulator: mixed-methods protocol to test social network interventions for resilience, health, and well-being of Amsterdam adults

Authors

Kaan, Jiri; Ulug, Ciska; Thompson, Kristina; Khaluf, Yara; Wagemakers, Annemarie; Moore, Spencer

---

This article was not externally reviewed at BMJ Open. Protocols that have been independently assessed prior to submission to BMJ Open are usually fast-tracked to publication on the grounds that further substantial changes will not be possible. This independent assessment will usually be external, independent review for both a substantial grant award from a non-commercial or government funder and ethics approval.
